# Effect of redox reactions on the thermoluminescence characteristics of Cu-doped NaLi_2_PO_4_ phosphors

**DOI:** 10.1039/d3ra02498a

**Published:** 2023-07-06

**Authors:** Bhuli Bai, P. D. Sahare

**Affiliations:** a Department of Physics and Astrophysics, University of Delhi Delhi-110007 India pdsahare@yahoo.co.in pdsahare@physics.du.ac.in

## Abstract

A Cu-doped NaLi_2_PO_4_ phosphor material was successfully synthesized through the high-temperature solid state diffusion method. It was mainly doped with Cu_2_Cl_2_ and CuCl_2_ salts for impurities in the form of Cu^+^ and Cu^2+^, respectively. Formation of the material in the single phase of the phosphor material was confirmed by powder XRD. Morphological and compositional characterization was done using XPS, SEM and EDS techniques. The materials were annealed in reducing, (10% H_2_ in Ar) and CO/CO_2_ (by burning charcoal in a closed system), as well as in oxidizing (air) atmospheres at different temperatures. ESR and PL studies were conducted for studying redox reactions due to annealing and its effect on TL characteristics. It is known that the impurity Cu could exist in Cu^2+^, Cu^+^ and Cu^0^ forms. The material was doped with two different salts (Cu_2_Cl_2_ and CuCl_2_) as sources of the impurities in two different forms *i.e.*, Cu^+^ and Cu^2+^, however, it was found that it gets incorporated in both the forms inside the material. Also, annealing in different atmospheres not only changed their ionic states but also affected the sensitivity of these phosphors. It was observed that at ∼10 Gy, NaLi_2_PO_4_:Cu(ii) is around 3.3 times, 3.0 times and almost equally sensitive than commercially available TLD-900 phosphor on annealing in air, 10% H_2_ in Ar and CO/CO_2_ at 400, 400 and 800 °C, respectively. However, NaLi_2_PO_4_:Cu(i) becomes 1.8 times sensitive after annealing in CO/CO_2_ at 800 °C as compared to TLD-900. With high sensitivity, both the materials NaLi_2_PO_4_:Cu(ii) and NaLi_2_PO_4_:Cu(i) are good candidates for radiation dosimetry with a wide dose response (mGy–5.0 kGy).

## Abstract 2

Change in colour after annealing at different temperatures: (a and d): NaLi_2_PO_4_:Cu(ii) and NaLi_2_PO_4_:Cu(i) in air, (b and e) NaLi_2_PO_4_:Cu(ii) and NaLi_2_PO_4_:Cu(i) in 10% H_2_, (c and f) NaLi_2_PO_4_:Cu(ii) and NaLi_2_PO_4_:Cu(i) in CO/CO_2_, respectively.

## Graphical abstract

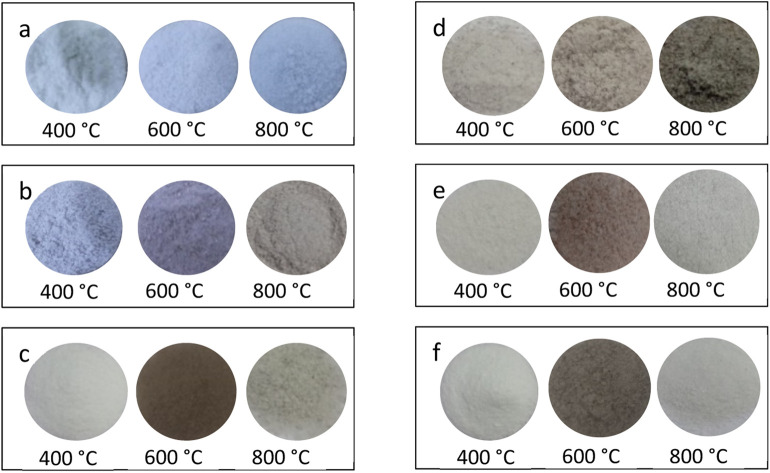

## 1. Introduction

Thermoluminescence (TL) is one of the popular dosimetric techniques used for measuring doses of high energy-radiations. In this technique, electron traps and holes (luminescence centers) are generated during irradiation which could be stimulated by providing heat energy. The energy of recombinations emitted as TL on stimulation in the form of visible light makes it possible to estimate the doses of high-energy radiations as the intensity of the emitted light is proportional to the absorbed doses.^1–4^ In search of an “ideal dosimeter”, many TLD phosphors have been investigated. But most of them still possess one or more drawback(s).^5,6^ A good TLD material should consist of the following ‘good’ characteristics, such as, simple synthesis process, low-*Z* (tissue equivalence), high sensitivity, non-toxicity, negligible fading, linearity over a wide dose range and excellent reusability. NaLi_2_PO_4_ doped with rare earth impurities, is a well-known highly sensitive TLD/OSLD phosphor material.

Initially, Shinde and Dhoble in 2011 reported reddish-orange emission for Eu^3+^ and Tb^3+^-doped NaLi_2_PO_4_ materials for white-LED applications.^7,8^ Later, in 2014 Sahare and Singh observed intense red emission with 99.9% colour purity due to peak wavelength centered at around 702 nm in the former phosphor.^9^ Further, PL emission due to Eu^2+^ ion in blue region, because of induced reduction of Eu^3+^ on irradiation with γ-rays, was also observed.^10^ Sahare and co-workers further explored rare earth impurity(ies) (Eu^3+^, Ce^3+^, Tb^3+^) doped NaLi_2_PO_4_ materials for their applications as TLD and OSLD phosphors.^11–15^ However, rare-earth materials are quite expensive and some of them may consist of radioactive isotopes that may give self-dose introducing errors in dose estimations, especially, at low dose levels. Other transition metals can also act as luminescence activators, such as Mn^4+^,^16^ Ti,^17^ Cr^3+^ ^18^ or Cu^19–30^ for such hosts. For example, LiF:Mg,Cu,P^21–23^ and Li_2_B_4_O_7_:Cu,^24^*etc.* are well-known dosimetry phosphors. PL, TL and OSL in some Cu-doped lithium based-orthophosphates have also been reported.^25–29^ This prompted us to study Cu-doped NaLi_2_PO_4_. We were more encouraged when we saw that the phosphor material is many fold sensitive than the commercially available popular material CaSO_4_:Dy (TLD-900) procured from Nucleonics Systems Pvt. Ltd Hyderabad, India.

Recently, Sahare *et al.*^30^ studied Cu-doped LiF nanophosphor. Their interests seem to be investigating the role of Cu impurity in well-known LiF:Mg,Cu,P phosphor on heating/annealing on TL readouts beyond 250 °C. It was important as the phosphor loses its reusability on heating beyond 250 °C and if not heated some remaining traps could add errors during its reuse. They could reveal that this is because of the incorporation of the impurity in both Cu^2+^ and Cu^+^ ionic forms though doping is done with either of the starting materials (*i.e.*, CuCl_2_ or Cu_2_Cl_2_). They also further showed using different experimental techniques, such as, TL, ESR, PL that redox reactions taking place during irradiation/readouts/annealing, *etc.* And all the traps might not either be emptied as the material is not heated beyond 250 °C or all the impurity ions in different states after irradiation might not be coming back to their initial states on readouts/annealing. According to them, if this is occurring in Cu-doped LiF and reusability is not maintained, it must be occurring in LiF:Mg,Cu,P also. This was another reason to take up this study to see whether such reactions are also taking place in Cu-doped NaLi_2_PO_4_ material as well. It has lot of significance from application point of view, if it is found occurring in many Cu-doped phosphors, then their applications as TLD/OSLD/white LED/lamp phosphors would be questionable.

In the present work, Cu-impurity was tried as an activator in NaLi_2_PO_4_ for its application as TLD phosphor. The material was doped with Cu_2_Cl_2_ and CuCl_2_ salts as starting impurities in Cu^+^ and Cu^2+^ ionic forms, respectively. However, it was found that it gets incorporated in both the forms inside the material. It may be noted here that though the doped impurity gets incorporated in to two ionic forms, *i.e.*, Cu^+^ and Cu^2+^, there is no evidence that they act as activators and sensitizers. For optimizing the luminescence characteristics, the materials were annealed in reducing (10% H_2_ in Ar and CO/CO_2_ atmosphere by burning charcoal in a closed system) and oxidizing (air) atmospheres at different temperatures. The redox reactions were studied and found useful for optimizing the dosimetry properties. High sensitivity (around 2.2 times at low doses and 6.8 times at high doses compared to that of TLD-900) in the wide dose range of mGy–kGy, excellent reusability, *etc.* makes the phosphor a good candidature for dosimetry of high energy radiations. Effective atomic number (*Z*_eff_) of the material was determined by using the formula 
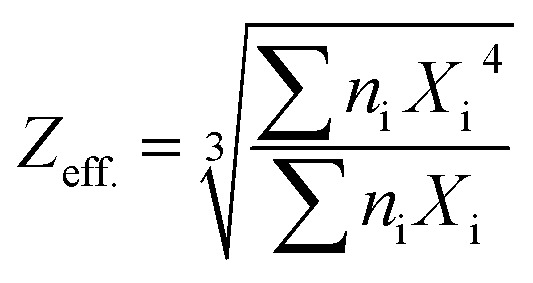
, where, *n*_i_ is the fractional part by weight of whole compound occupied by an element i, *X*_i_ is the atomic numbers of that element.^31^ It was found to be 10.8 for NaLi_2_PO_4_.

## 2. Experimental

### 2.1. Synthesis

Microcrystalline form of Cu^2+^ doped NaLi_2_PO_4_ phosphor prepared by high temperature solid state method. Details could be found in our earlier papers.^11–15^ It has also been described here for beginners. The initial materials LiOH·H_2_O and NaH_2_PO_4_·H_2_O (99.0% Central Drug House, India). The materials were prepared taking into the consideration the following chemical reactions:
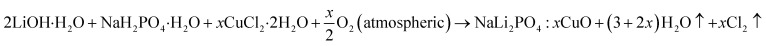

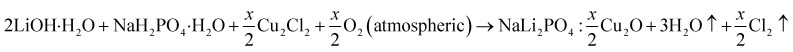
where, *x* is the dopant concentration.

Initially, suitable amount of CuCl_2_·H_2_O (CDH, India) and Cu_2_Cl_2_ (CDH, India) were taken as sources of Cu^2+^ and Cu^+^ impurity ions, respectively. It is to be noted here that one needs to be little cautious while calculating the impurity concentrations as the former consists of one Cu^2+^ ion while the later consists of two Cu^+^ ions. The material doped with the salt having Cu^+^ initial ionic state has been named as NaLi_2_PO_4_:Cu(i) and the other one doped with the salt having Cu^2+^ initial ionic state was named as NaLi_2_PO_4_:Cu(ii). LiOH·H_2_O and NaH_2_PO_4_·H_2_O taking into molar ratio of 2 : 1 along with the required amount of impurity salt were thoroughly mixed using an agate motor and pestle continuously for few hours in the presence of ethanol for better mixing. The mixture in powder forms was initially heated for 12 h at 400 °C in quartz container in a resistive furnace controlled by a temperature-controller and programmer (West 6400 with stability around ±1.0 K) and cooled slowly to room temperature (RT). The material (ingot), thus formed was crushed to fine particles and given heat treatment at 600 °C and repeated the same at 800 °C for the same interval of time and again cooled down slowly to RT. The sample thus prepared was finally crushed in motor pestle and sieved (by using standard test sieves) to obtaine different particle size ranges. The best results were obtained for the particle size in the range of 63–150 μm of NaLi_2_PO_4_:Cu(ii). All the prepared samples were annealed in oxidizing atmosphere (air) at different elevated temperatures ranging from 100 °C to 1000 °C for 1.0 h and quenched to RT by placing quartz container on a metal block to improve sensitivity. These materials were used for further studies.

### 2.2. Annealing in reducing atmospheres

The annealing of the samples in reducing atmospheres (10% H_2_ in Ar and also in CO/CO_2_ by burning charcoal in a closed system) was also done. For annealing in 10% H_2_ in Ar gas the following procedure was adopted. Initially, the samples were put in alumina boat inside a cylindrical alumina tube inserted in a tubular furnace. As mentioned earlier, the temperature of the furnace was controlled by a temperature controller and programmer. One end of the alumina tube was connected to gas cylinder (filled with 10% H_2_ in Ar for generating reducing atmosphere) through a digital mass flow controller (DMFC) and the other end was attached to a gas bubbler through which gas could flash out into the atmosphere. The samples were put inside at the centre of the tubular furnace and the desired temperatures were reached with the help of temperature controller at the rate of 100 °C h^−1^. The annealing was carried out at different temperatures, such as, 400, 600 °C and so on for 1.0 h. After annealing the samples were cooled to room temperature naturally under the same atmosphere by switching of the furnace. The same treatment was given to a set of all the samples for further studies.

Another set of samples was also annealed in CO/CO_2_ (reducing) atmosphere by burning charcoal in a closed system. For this, samples were placed in alumina crucibles and covered using ceramic wool under a steel net and another alumina crucible one above the other and upside down (in order to avoid contamination of the sample with the fine charcoal powder while heating).^32,33^ These crucibles were then placed inside a closed charcoal box. Burning of the charcoal under scarcity of oxygen produces CO/CO_2_ gas as reducing atmosphere. Annealing of samples under this atmosphere at different temperatures, *i.e.*, 400 °C, 600 °C and 800 °C, *etc.* for 1.0 h was done by controlling and maintaining the furnace temperature as mentioned earlier.

### 2.3. Characterization and measurements

The powder X-ray diffraction (PXRD) patterns were recorded at room temperature using a high-resolution Bruker D8 X-ray diffractometer and utilizing its monochromatic Cu_Kα_ line (*λ* = 1.54060 Å) radiation (*E* = 8.04 keV) with scan rate 2° min^−1^, step dimension of 0.02 s and 2*θ* ranging from 20° to 80°. The PXRD diffractometer was operated at a voltage of 40 kV and current 25 mA. TL were taken under nitrogen atmosphere after irradiating the material with γ-rays for different doses from ^60^Co radioactive source (gamma chamber) using the 3500 Harshaw TLD Reader attached with PMT as detector and temperature programmer for linear heating by taking ∼5 mg of sample each time. The chemical composition and elemental analysis were done using XPS technique (Thermo Fisher Scientific, UK, Model: K-Alpha XPS) with energy resolution of 0.1 eV at a base vacuum of 10^−9^ Torr. XPS peaks were deconvoluted using the Peak-41 Software. Free radicals and transition metal ions can be identified using ESR technique. ESR was done ESR instrument (Bruker Biospin, Germany, Model: EMXmicro A200-9.5/12/S/W) with microwave X band source. The morphology of the materials annealed in different atmospheres was studied by high resolution images using SEM instrument (JEOL, Japan, Model: JSM 6610LV). PL emission and excitation spectra was taken with the help of Horiba Scientific fluorescence spectrophotometer, PTI QM-8450-11-C system # 3560.

## 3. Result and discussion

### 3.1. PXRD analysis

**Fig. 1:**
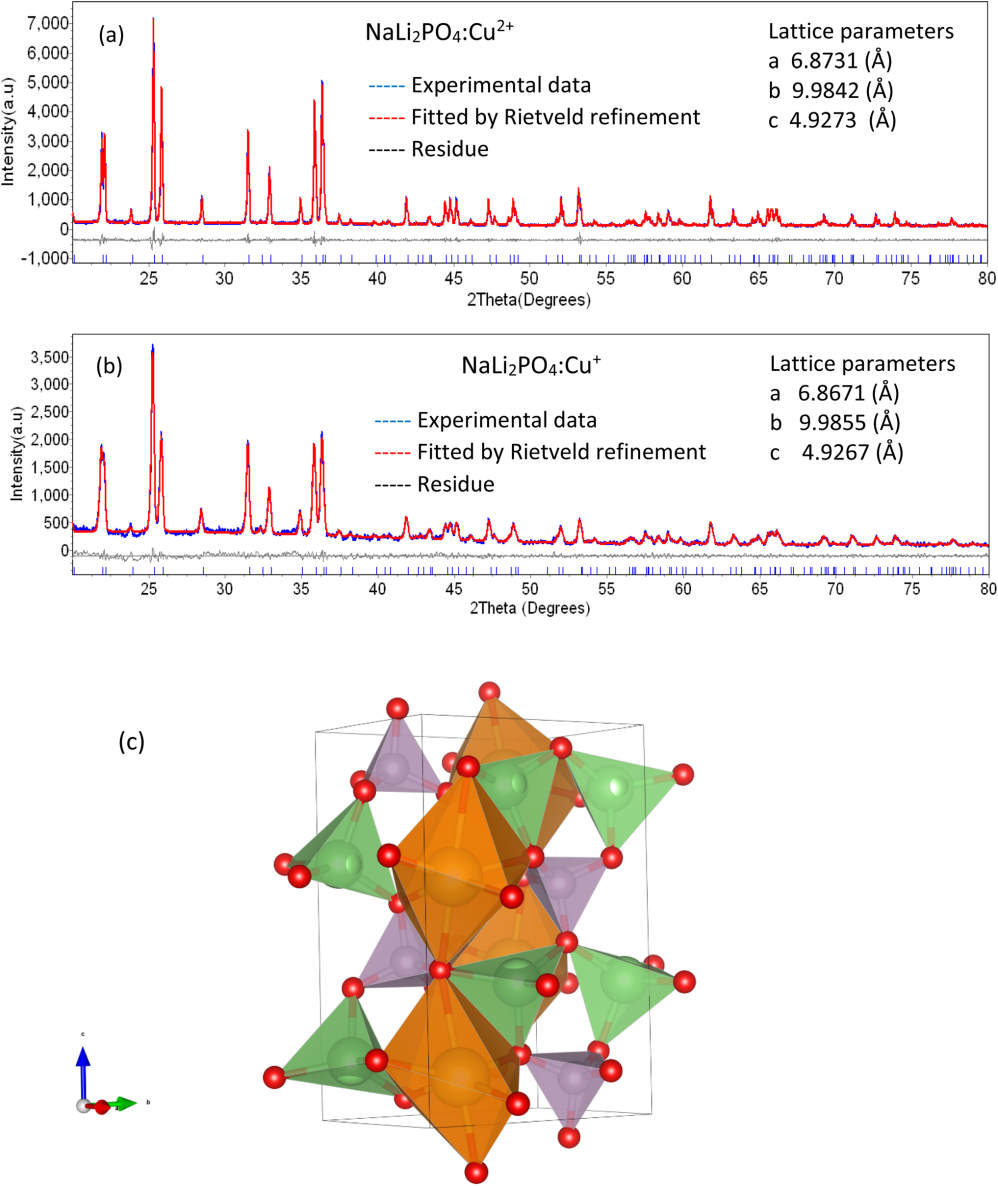
PXRD patterns of the NaLi_2_PO_4_ phosphors: (a) PXRD pattern of the NaLi_2_PO_4_:Cu(ii) phosphor material, (b) PXRD pattern of the NaLi_2_PO_4_:Cu(i) phosphor material, (c) crystal structure. Theoretically fitted by Rietveld refinement patterns along with JCPDS data (file # 80-2110) are shown for comparison. The lattice parameters used for the fittings are also shown in the inset.

The PXRD patterns of the microcrystalline NaLi_2_PO_4_:Cu(i) and that of NaLi_2_PO_4_:Cu(ii) phosphor materials were matched with that of the reported data in the literature PCPDF file # 80-2110 with lattice parameter *a* = 6.884, *b* = 9.976, *c* = 4.927 and *α* = *β* = *γ* = 90°. It was also confirmed that both the materials have single phase having space group *Pmnb*(62) and orthorhombic structure. The experimental data was also analysed using Rietveld refinement. It could be seen from Fig. 1(a) and (b) that the experimental and theoretically fitted data matches well with each other and also with the standard JCPDS file # 80-2110. No additional peak(s) related to impurity or any other phase was present. It could be seen that the lattice parameters also match.

The 3D-crystal structure has been shown in Fig. 1(c). As seen in the figure, Na^+^ is bonded to six O^2−^ ions in a 6-coordinated geometry and the Na–O bond distances range from 2.30–2.82 Å, while Li^+^ forms LiO_4_ tetrahedra and is bonded to four O^2−^ ions to share corners with four equivalent LiO_4_ tetrahedra. The Li–O bond distances range from 1.98–2.02 Å. P^5+^ ion is bonded to four O^2−^ ions to form PO_4_ tetrahedra that share corners with eight equivalent LiO_4_ tetrahedra. All P–O bond lengths are 1.56 Å. Thus, there are three inequivalent O^2−^ sites as shown in the figure. In the first O^2−^ site, O^2−^ is bonded in a 5-coordinate geometry to two equivalent Na^+^ ions, two equivalent Li^+^ ions and one P^5+^ ion. In the second O^2−^ site, O^2−^ is bonded to one Na^+^ ion, two equivalent Li^+^ ions and one P^5+^ ion to form distorted corner-sharing ONaLi_2_P trigonal pyramids. In the third O^2−^ ion site, O^2−^ ion is bonded in a 5-coordinated geometry to two equivalent Na^+^, two equivalent Li^+^ and one P^5+^ ion.^34^

The ionic radii of Li^+^, Na^+^, P^5+^, O^2−^, Cu^2+^, Cu^+^ and Cu^0^ are 0.76, 1.02, 0.38, 1.40, 0.73, 1.40, and 1.45 Å, respectively. From this data, it could be seen that the ionic radii of Li^+^ and that of Cu^2+^ are 0.76 and 0.73 Å, respectively, therefore, two Cu^2+^ ions could go to the two Li^+^ substitutional sites and the oxygen (O^2−^) ion (diffused from the atmosphere) at the interstitial site do the charge compensation by forming bonds with Cu^2+^ ions. But, when it comes to Cu-impurity in its Cu^+^ ionic form, the ionic radii neither match that of Li^+^ nor that of Na^+^. Thus, it does not seem to be possible to substitute any of these two ions, moreover, it could neither go to P^5+^ site as the radius is too small. Therefore, it could get incorporated at the oxygen vacancies (revealed in XPS analysis) as the ionic radius of (O^2−^) ion/its vacancy being the same as that of Cu^+^ ion (1.40 nm) or go to interstitial site. The oxygen vacancy(ies) could exist in (O_v_^2+^), (O_v_^+^) and (O^0^_v_) forms^35^ by sharing electron pairs of other oxygen ions attached to the PO_4_^3−^. Thus, two Cu^+^ ions (both may be oxygen vacancy occupied ions at nearby PO_4_^3−^ tetrahedra or at interstitial sites) could form bonds with the oxygen (O^2−^) ion at the interstitial site (diffused from the atmosphere) and compensate charges. On annealing in reducing/oxidising atmospheres the ratio of Cu^2+^, Cu^+^ and/or that of oxygen vacancies could change keeping the material neutral. This is confirmed by XPS and ESR analysis discussed below (Sections 3.7 and 3.8).

### 3.2. SEM/EDS analysis

**Fig. 2:**
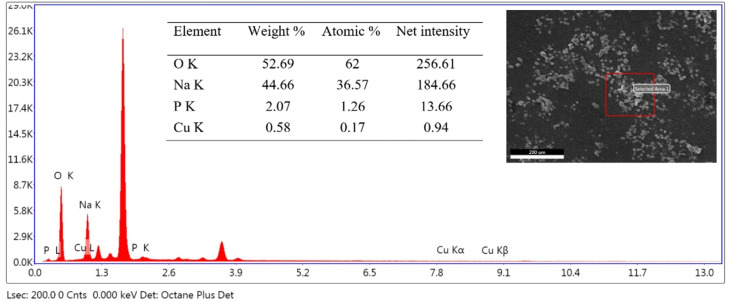
A typical EDS spectrum of NaLi_2_PO_4_:Cu(ii) (0.1 mol%) material. The SEM image and the elemental composition are also shown in the inset.

SEM and EDS analysis of the materials were done to study the morphology and composition of the materials. A typical EDS spectrum of NaLi_2_PO_4_:Cu(ii) (0.1 mol%) is shown in Fig. 2. SEM image and a table of the composition of the constituent elements are also shown in the insets. It could be seen in the figure that well-defined granular particles are seen in the SEM image. The Cu impurity concentration (0.17%) is of the same order of the doping.

### 3.3. Effect of impurity concentration on TL properties of Cu-doped NaLi_2_PO_4_

**Fig. 3:**
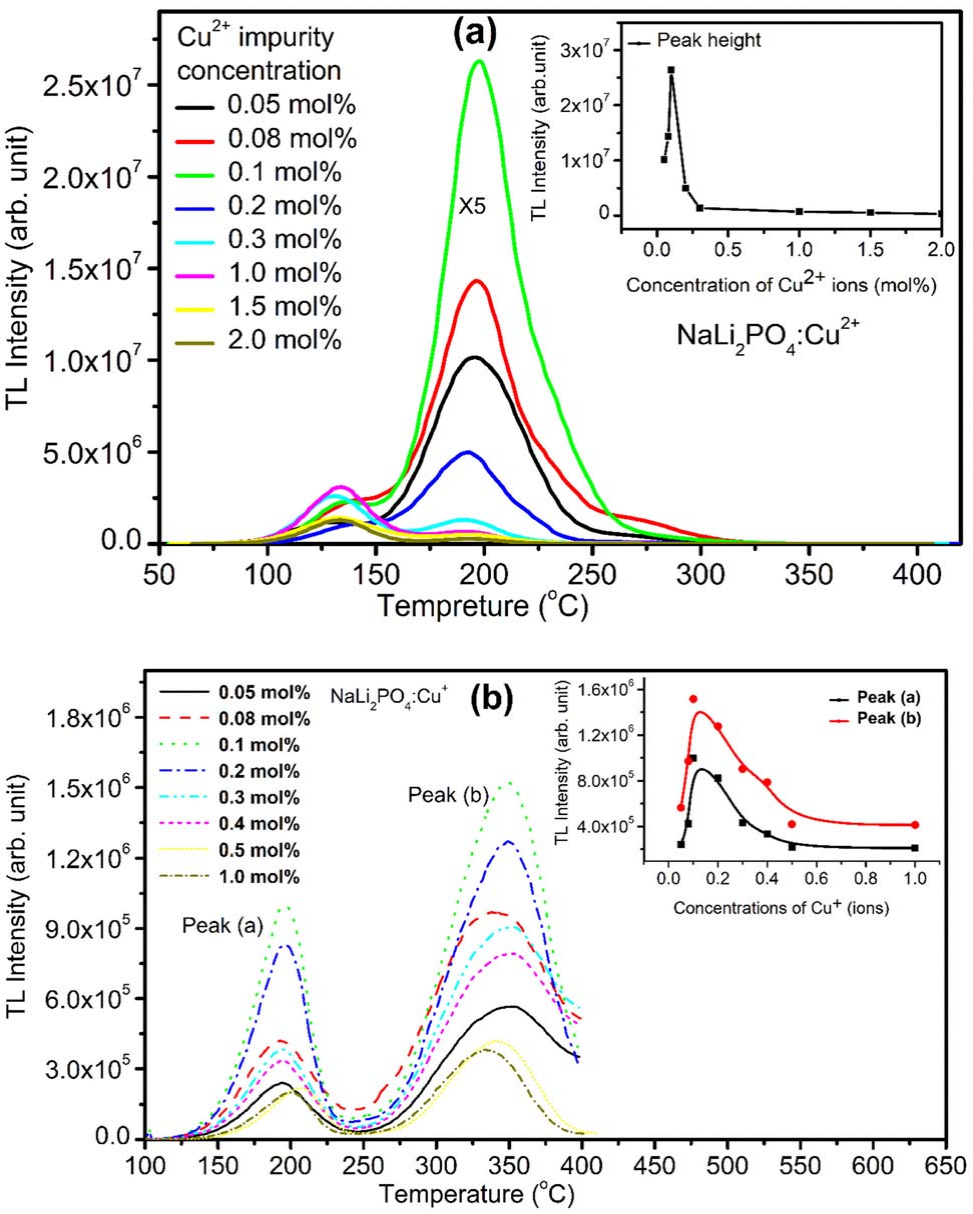
(a) Optimization of concentration of the impurity in the host matrix NaLi_2_PO_4_. TL glow curve of the material doped with different impurity (Cu^2+^) concentrations (0.05–2.0 mol%) and exposed at 10 Gy of γ-rays from Co^60^ source. The materials were annealed at 400 °C in air. Inset shows TL intensity of NaLi_2_PO_4_:Cu(ii) *vs.* Cu^2+^ impurity concentration, (b) TL glow curve of the material doped with different impurity (Cu^+^) concentrations (0.05–1.0 mol%) and exposed at 10 Gy of gamma-rays from Co^60^ source. Inset shows TL intensity of NaLi_2_PO_4_:Cu(i) *vs.* Cu^+^ impurity concentration.

Variation of impurity concentration (0.05–2.0 mol%) with TL intensity of NaLi_2_PO_4_:Cu(ii) is shown in Fig. 3(a). The materials were annealed at 400 °C in air. It was observed that on increasing the doping concentration, TL intensity increases up to 0.1 mol% after that it starts decreasing due to concentration quenching effect. However, there are some small changes in the glow curve structures at high concentrations which may be attributed to the rearrangement of trapping levels originated due to incorporation of the impurity in different ionic states, *i.e.*, Cu^+^ and Cu^2+^. The impurity could be incorporated in the form of Cu^+^ and Cu^2+^ ionic states, though the starting ionic state was in Cu^2+^ form.^30,36^ But the ratio of Cu^+^/Cu^2+^ ions may vary with the impurity concentration. As discussed earlier, Cu^2+^ ions may go to substitutional sites of Li^+^ ions and the charge compensation is done the oxygen ions diffused from the air atmosphere. This may, however, change the TL sensitivity of the material. The maximum TL intensity at doping concentration (Cu^2+^ ions) is 0.1 mol% observed at 10.0 Gy Co^60^ source.

For NaLi_2_PO_4_:Cu(i) material doped with Cu^+^ impurity, the maximum TL intensity was found at 0.1 mol% shown in Fig. 3(b). The materials were annealed at 400 °C in air. But, as mentioned earlier, it is not necessary that the impurity would be incorporated as Cu^+^ only even if it is in this form initially in the salt used for doping, it may be incorporated in both Cu^+^ as well as in Cu^2+^ forms.^30,36^ It is also known that the TL intensity of Cu^+^ (using Cu_2_Cl_2_ salt) initially doped material is more than that of Cu^2+^ (using CuCl_2_ salt), for example, Cu-doped LiF.^30^ However, it seems here that it is otherwise, *i.e.*, the TL intensity of the Cu^2+^ initially doped material is more than that of the Cu^+^ doped. This may be because of difference in their ionic radii (0.73 Å for Cu^2+^ and 1.40 Å Cu^+^, respectively). As the ionic radius of Cu^2+^ is much comparable with that of Li^+^ (0.76 Å) while that of Cu^+^ is almost the same that of O^2−^ (1.40 Å). Also, it is easier for charge compensation of the additional charges of the Cu^2+^ ions by forming bonds with the nearby O^2−^ions diffused from air atmosphere. More studies on redox reactions after giving annealing treatments at different temperatures and in different oxidizing and reducing atmospheres has been done and discussed in next sections (Sections 3.3–3.5). In both the cases, however, as the impurity concentration increases, it results in concentration quenching.

A number of processes may be involved in concentration quenching, such as, interaction between the quencher and the activator (both could be same kinds of ions/radicals or may also be different), energy transfer, charge transfer reaction or complex formation. Quenching could be a static or dynamic phenomena. The dynamic quenching could be attributed to the energy transfer between activators, which come closer to each other with increase in concentration and agglomeration. This results in increase of probability of the non-radiative interaction due to tunnelling.^37^ However, in the present case, it would be more complex as it is not just the energy transfer between the quencher and the activator but it is due to electron traps (trapped at the local energy levels of the activators) and holes/luminescence centres (LCs) generated on irradiation.

On stimulation by heat energy in TL, the traps make several attempts for detrapping (frequency factor) as the stimulation energy is very less (*k*_B_*T* in meV) than the trap depth (energy difference the trap level and the lowest level of the conduction band which is of the order of a few eV). They could get re-trapped also. As there are three kinds of activators (Cu^2+^, Cu^+^ and Cu^0^) and redox reactions could occur on irradiation and annealing, it is difficult to say which mechanism would be responsible for concentration quenching. However, mostly nonradiative recombinations of the shallow traps, crossover energy level transitions, retrapping and more complex defect formations at higher concentrations would be responsible for quenching.

### 3.4. Effect of annealing temperatures on TL glow curves and intensity after annealing in oxidizing atmosphere (air)

**Fig. 4:**
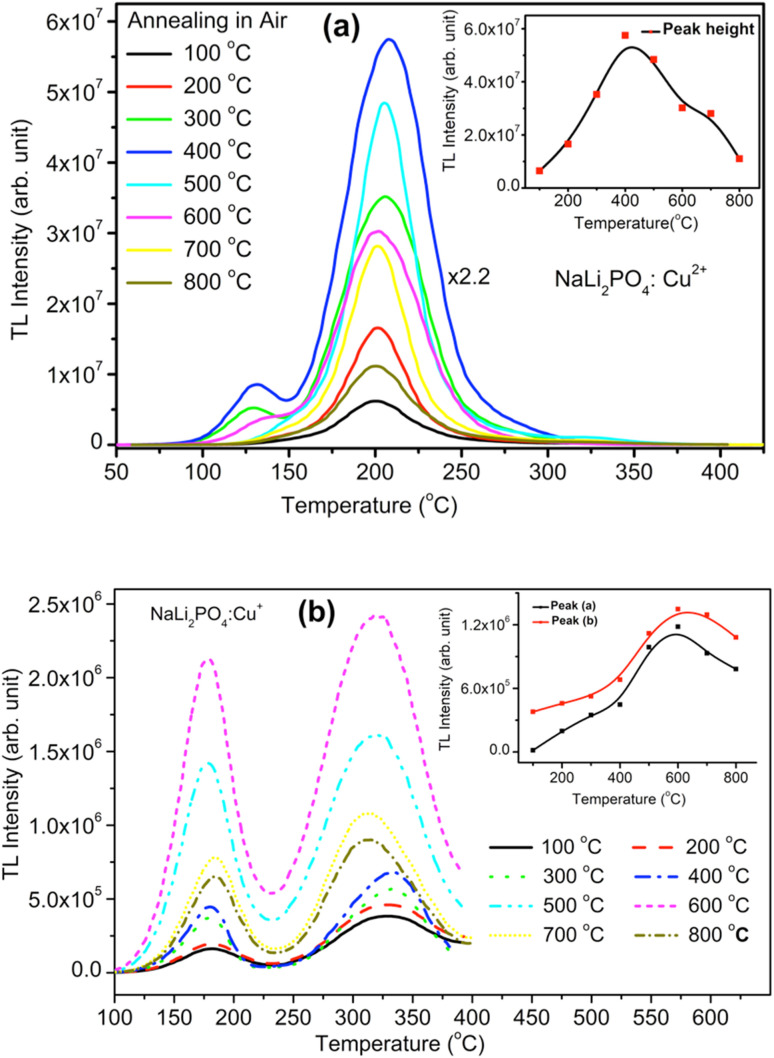
(a) Optimization of the annealing temperature effect on NaLi_2_PO_4_:Cu(ii). TL glow curve of the material with doped Cu^2+^ impurity (0.1 mol%) concentration and exposed at 10 Gy of gamma-rays from the Co^60^ source. The plot of TL intensity (peak height) *vs.* annealing temperature is also shown in the inset, (b) optimization of the annealing temperature effect on NaLi_2_PO_4_:Cu(i). TL glow curve of the material doped with Cu^+^ impurity (0.1 mol%) concentration and exposed at 10 Gy of gamma-rays from the Co^60^ source. The plot of TL intensity (peak height) *vs.* annealing temperature is also shown inside.

NaLi_2_PO_4_:Cu(ii) material (0.1 mol%) was annealed in air at different elevated temperatures (ranging from 100–800 °C). Effect of annealing temperature is shown in Fig. 4(a) where it could be observed that 400 °C is optimized temperature for highest intensity. Similarly, NaLi_2_PO_4_:Cu(i) material (0.1 mol%) was annealed at different elevated temperatures (ranging from 100–800 °C), as the annealing temperature is increased, the TL intensity of the dosimetric peak increases up to 400 °C but it starts gradually decreasing on annealing beyond this temperature. Annealing temperature effect for NaLi_2_PO_4_:Cu(i) is shown in Fig. 4(b) and it could be seen that optimized temperature to achieve the highest TL sensitivity for NaLi_2_PO_4_:Cu(i) is around 600 °C. There are reports that atmospheric oxygen could get diffused inside the material on annealing at higher temperatures in air and may help propagating redox reactions of the impurity of Cu-ions.^38,39^ The TL sensitivity goes on increasing with the annealing temperature increasing till 600 °C (at which TL intensity was maximum) but starts decreasing thereafter. The possible reason for this could be the more uniform dispersion of impurity ions in the crystal but further decrease in intensity (*i.e.*, beyond 600 °C) could be attributed to diffusion of atmospheric oxygen leading to more complex defects at the impurity ion sites as well as redox reactions.^32,40^

It is known that after heat treatments above 250 °C, the ratios of TL emissions 380 and 350 nm corresponding to Cu^2+^ and Cu^+^ in Cu-doped LiF, respectively, in the samples annealed at different temperatures decrease; this has been attributed to the Cu^+^ → Cu^2+^ + e^−^ conversions during heating treatments.^32,40^ Similar results are also observed by Yang *et al.* in Cu-doped LiF^41^ on irradiation to different γ-ray doses and it has been shown that the relative intensity of the two peaks (appearing at around 187 and 292 °C) in Cu-doped LiF change again due to Cu^+^ → Cu^2+^ conversions. Apparently, there are two TL glow peaks, one at around 127 °C and another at around 202 °C were observed in NaLi_2_PO_4_:Cu(ii) and 177 and 327 °C in NaLi_2_PO_4_:Cu(i) materials. We also have observed the change in peak ratios of NaLi_2_PO_4_:Cu(i) and NaLi_2_PO_4_:Cu(ii) materials annealed in air. This may be attributed to the redox reactions taking place in these materials. At high-temperatures, such redox reactions could also occur in reverse order, *i.e.*, Cu^2+^ + e^−^ → Cu^+^.^30^ We strongly believe that such conversions must be taking place in our Cu-doped NaLi_2_PO_4_ materials also. However, it is difficult to assign a particular TL glow peak(s) to one or the other kind(s) of impurity(ies) in a phosphor material as the TL emissions could take place from different kinds of traps competitively at the same time and even if it is not occurring at the same time a number of traps could move from deeper traps to shallow traps at their positions after their thermal stimulation and recombinations.

### 3.5. Effect of annealing temperatures on TL glow curves and intensity after annealing in different reducing atmospheres (H_2_ in Ar and CO/CO_2_)

**Fig. 5:**
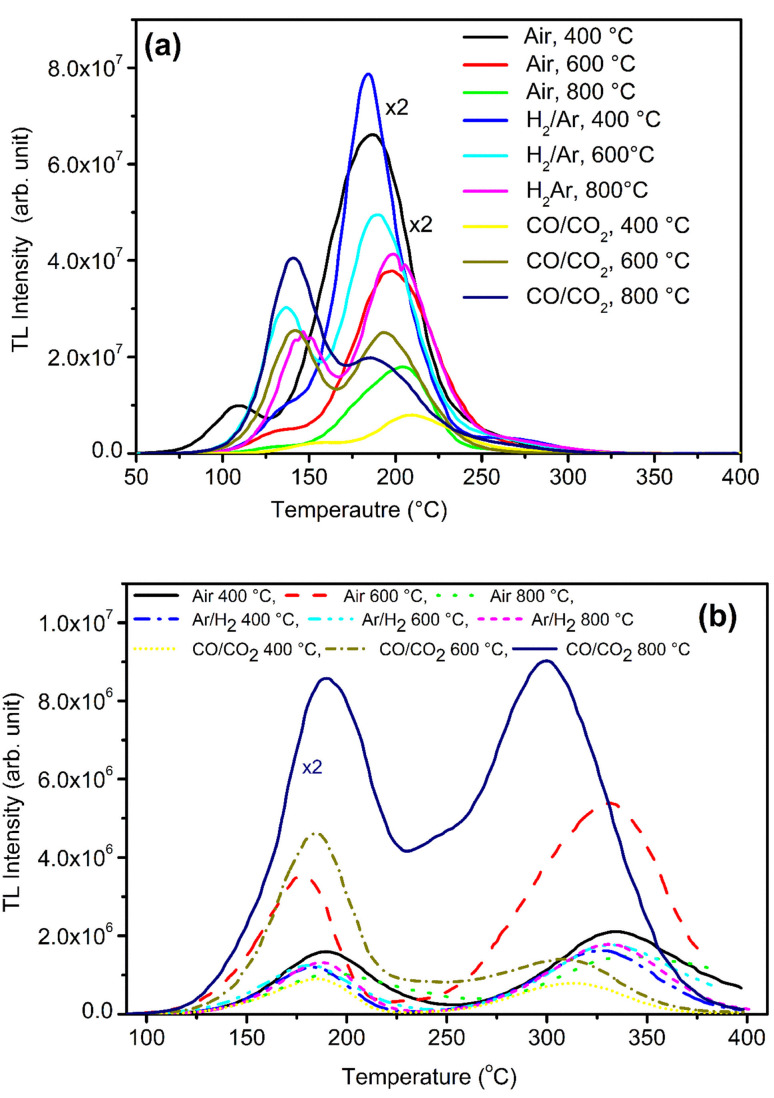
(a) NaLi_2_PO_4_:Cu(ii) phosphor material annealed in various atmosphere at different temperatures 400, 600 and 800 °C for 1.0 h, (b) NaLi_2_PO_4_:Cu(i) phosphor material annealed in various atmosphere at different temperatures 400, 600 and 800 °C for 1.0 h.

Samples were annealed in H_2_ atmosphere (10% H_2_ in Ar) at different temperatures (*i.e.*, 400, 600 and 800 °C) using the procedure described earlier (Section 2.2). NaLi_2_PO_4_:Cu(ii) was annealed in hydrogen atmosphere at 400, 600 and 800 °C for 1.0 h. After annealing TL of these materials was taken. The results are as shown in Fig. 5(a). The TL glow curves apparently consist of two peaks, one at around 137 and another at around 177 °C. There is a significant change in the peak temperatures (∼25 °C) and change in their intensity ratios on annealing at different temperatures and in different atmospheres show that some redox reactions due to heating, such as, Cu^2+^ + e^−^ → Cu^+^ + e^−^ → Cu^0^ might be occurring inside the material.^30,36^ However, the number of such different species may also change due to redox reactions on irradiation.^41^ The TL intensity was found to be maximum for annealing at 400 °C due to reduction of Cu^2+^ + e^−^ → Cu^+^. However, further increase in temperature may result in further reduction of the impurity in metallic copper, *i.e.*, Cu^+^ + e^−^ → Cu^0^ leading to quenching of the TL intensity.

Similar, type of behavior was observed on annealing in CO/CO_2_ atmosphere (Fig. 5a) NaLi_2_PO_4_:Cu(ii) samples were annealed at 400 °C, 600 °C, 800 °C in charcoal atmosphere for 1.0 h then TL was taken. The intensity of the TL glow peaks annealed in this (CO/CO_2_) atmosphere is very less than the same annealed in hydrogen atmosphere at the same temperatures, *i.e.*, 8, 6.0 and 2.2 times at 400 °C, 600 °C, 800 °C, respectively. There is also a reverse trend than observed in case of the material annealed in 10% H_2_ in Ar gas atmosphere, *i.e.*, the intensity is lowest for the sample annealed in CO/CO_2_ atmosphere at 400 °C and increases subsequently on annealing at 600 °C and saturates thereafter. This may be occurring because of two reasons. Firstly, as the CO/CO_2_ is stronger reducing reagent than that of H_2_, most of the Cu^2+^ ions might have been reduced to metallic copper, *i.e.*, Cu^2+^ + e^−^ → Cu^+^ + e^−^ → Cu^0^. As mentioned earlier, it could be observed that the TL due to the impurity in Cu^2+^ ionic state is more than that of Cu^+^ state and may be least due to Cu^0^. This may be occurring due to changes in the relative ratios of these ionic species on irradiation.^30^

Study on effect of annealing was also done on NaLi_2_PO_4_:Cu(i) as well in order to understand its behavior in different atmospheres at high temperatures. Fig. 5(b) shows the variation in the TL glow curves of NaLi_2_PO_4_:Cu(i) with increase in temperature range 400 to 800 °C in different atmospheres for 1.0 h. Here also the TL glow curves apparently consist of two peaks one at around 177 and another at 327 °C. However, the change in peak temperatures on annealing at different temperatures in different atmosphere shows that there must be some redox reactions taking place in the material. It could be seen in the figure that the overall TL intensity of the material annealed in 10% H_2_ in Ar atmosphere is less than that of the material annealed in air and moreover, there is not much change on annealing at different temperatures. It seems that as H_2_ is a weak reducing agent than CO/CO_2_, it might not have reduced the Cu^+^ ions further to metallic Cu^0^ ions. But when this material was annealed in CO/CO_2_ atmosphere, most of the Cu^+^ ions might have been converted Cu^0^ ions which on irradiation could generate more Cu^+^ and electron traps. There are reports that diffusion of CO/CO_2_ in the material could generate more complex defects as in case of Al_2_O_3_:C and make the material more sensitive.^42,43^ The shift in the main dosimetry peak from ∼327 to ∼302 °C (∼25 °C) indicates generation of different kinds of traps related to such defects.

However, it needs to be seen that what kind traps are generated corresponding to different ions/radicals as well as holes (luminescence centers, LCs) formed during irradiation and whether their recombinations during TL readout is radiative or nonradiative. However, to understand the phenomenon in more details, we have taken ESR, XPS and PL measurements and are discussed in the following sections (Sections 3.7–3.9).

### 3.6. Change in colours of the images of powder samples on annealing in different atmospheres and temperatures

**Fig. 6:**
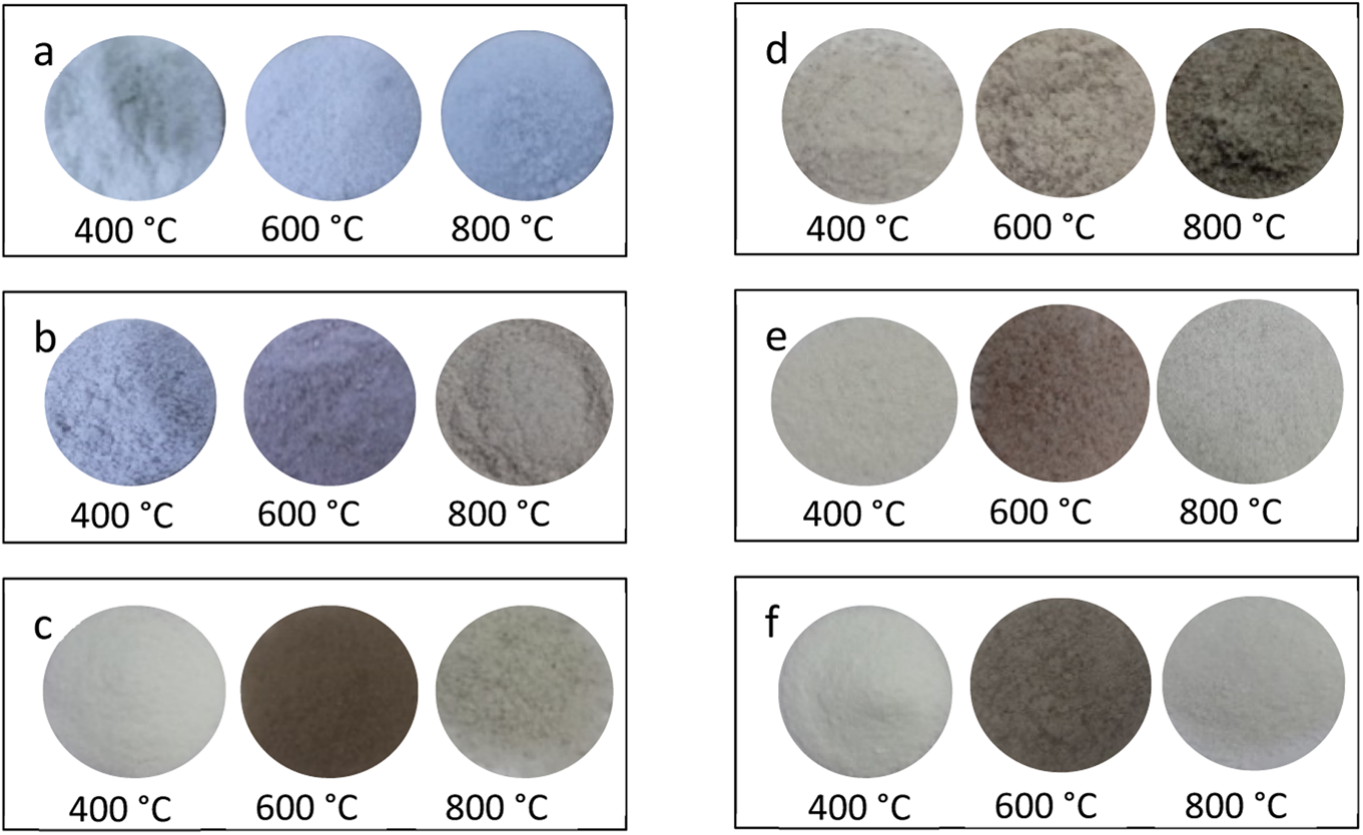
Change in colour of the powder samples after annealing: (a) NaLi_2_PO_4_:Cu(ii) annealed in air atmosphere at various temperatures, (b) NaLi_2_PO_4_:Cu(ii) annealed in 10% H_2_ in Ar atmosphere at various temperatures, (c) NaLi_2_PO_4_:Cu(ii) annealed in CO/CO_2_ atmosphere at various temperatures, (d) NaLi_2_PO_4_:Cu(i) annealed in air atmosphere at various temperatures, (e) NaLi_2_Po_4_:Cu(i) annealed in 10% H_2_ in Ar atmosphere at various temperatures, (f) NaLi_2_PO_4_:Cu(i) annealed in CO/CO_2_ atmosphere at various temperatures. All the images were taken in room light with high-resolution Canon DSLR camera EOS 1500D.

The changes taking place in the material (due to redox reactions) could also be visualized by change in the colour of the powder samples. The powder samples annealed at different temperatures in different reducing (*i.e.*, 10% H_2_ in Ar gas and CO/CO_2_ by burning charcoal in a closed system) atmospheres were spread on Petri dishes and their images were taken under room light. The images are as shown in Fig. 6(a) and (b). It could be seen in the figure that the changes in colors of these samples from bluish to grey and grey to brown, *etc.* in NaLi_2_PO_4_:Cu(ii) materials on annealing. Similar changes were also observed in NaLi_2_PO_4_:Cu(i) also except none of the samples were bluish. The change in colour of these materials clearly shows that some redox reactions were occurring in these materials.

### 3.7. ESR measurements

**Fig. 7:**
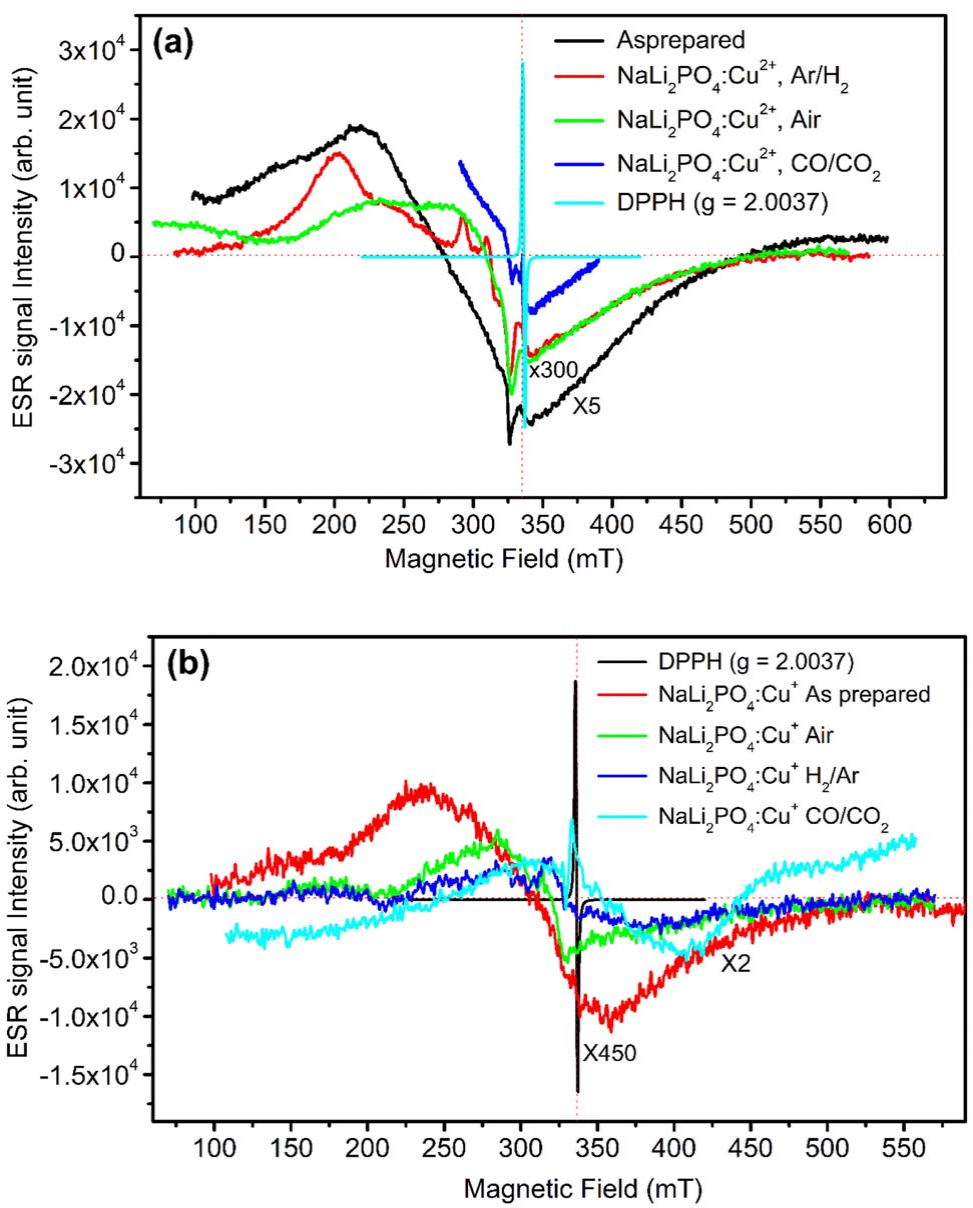
(a) ESR spectra of the microcrystalline NaLi_2_PO_4_:Cu(ii) phosphor materials annealed in air, 10% H_2_ in Ar and CO/CO_2_ atmospheres, (b) ESR spectra of the microcrystalline NaLi_2_PO_4_:Cu(i) phosphor materials annealed in air, 10% H_2_ in Ar and CO/CO_2_ atmospheres. The ESR spectrum for the DPPH (*g* = 2.0037) has also been given for calibration.

In order to understand more about the redox reactions in the materials under investigation, ESR measurements were taken at room temperature. As mentioned earlier, the materials were annealed in oxidizing atmosphere (air) and reducing atmosphere (10% H_2_ in Ar and CO/CO_2_) at different temperatures. However, the ESR spectra of the NaLi_2_PO_4_:Cu(ii) samples annealed at 400, 400 and 800 °C and that of NaLi_2_PO_4_:Cu(i) samples annealed at 600, 400 and 800 °C in air, H_2_ in Ar and CO/CO_2_ atmospheres, respectively, are given for better clarity. The spectra of as prepared (pristine) samples and that of 2,2-diphenyl-1-picrylhydrazyl (DPPH) are also given for comparison. The results are shown in Fig. 7(a) and (b) (Table 1). Lande *g*-factor was also determined using the following formula: *hν* = *μ*_B_*H*, where, *h* = 4.135 × 10^−15^ eV s, *ν* = 9.40 GHz, *μ*_B_ = 5.788 × 10^−5^ eV T^−1^, *H* = applied magnetic field (mT). The results are given in Table 2 and 3. The assignment of these values to different probable ions/radicals having unpaired electrons are also done. It may be seen in these tables that the values of *g*_∥_ and *g*_⊥_ vary on annealing in oxidising as well as reducing atmospheres showing that Cu^2+^ ions are in a very much stressed environments. Not only that but it is difficult to believe that in case of NaLi_2_PO_4_:Cu(i) material just on annealing in reducing, *i.e.*, CO/CO_2_ atmosphere at 800 °C, the TL intensity would increase many fold (Fig. 5(b)) as compared to that of annealed in air at 400 °C (optimized temperature for air atmosphere). The ESR spectrum in this case shows an ESR peak 324.5 mT indicating increase in the Cu^2+^ ions population as well as a new peak at around 340.4 mT, which could be assigned to CO_2_^−^ radicals.^44^ So, the carbon impurity thus incorporated may act as codoping and form new defects as in case of Al_2_O_3_:C and increase the sensitivity.^41,42^ In case of NaLi_2_PO_4_:Cu(i) also we have observed such peak, however, as the ionic state of the impurity (Cu^2+^) is different it might not have helped in enhancement of TL due to formation of different kinds of defects.

**Table 1:** Lande *g*-factors for NaLi_2_PO_4_:Cu(i) materials annealed in different atmospheres

Annealing atmosphere	Field (mT)	*g*_∥_	*g*_⊥_	Assignments
Pristine	230	2.924		Cu^2+^
	324		2.075	Cu^2+^
Annealed in air (600 °C)^a^	270	2.490		Cu^2+^
	324		2.075	Cu^2+^
Annealed in 10% H_2_ (400 °C)^a^	324		2.075	Cu^2+^
	327.5		2.054	Cu^2+^
	286.6	2.345		Cu^2+^
Annealed in CO_2_/CO (800 °C)^a^	324		2.075	Cu^2+^
	328	2.049		CO_2_^−^
	340		1.976	CO_2_^−^
DPPH (reference)	335.7		2.0036	

a Indicates annealing temperatures.

**Table 2:** Lande *g*-factors for NaLi_2_PO_4_:Cu(ii) materials annealed in different atmospheres

Annealing atmosphere	Field (mT)	*g*_∥_	*g*_⊥_	Assignments
Pristine	214.5	3.14		Cu^2+^
	324.5		2.073	Cu^2+^
Annealed in air (400 °C)^a^	231.5	2.906		Cu^2+^
	324.5		2.073	Cu^2+^
Annealed in 10% H_2_ (400 °C)^a^	261	2.580		Cu^2+^
	324.5		2.073	Cu^2+^
Annealed in CO_2_/CO (800 °C)^a^	300	2.242		Cu^2+^
	328		2.049	CO_2_−
	337		1.996	CO_2_^−^
	324.5		2.073	Cu^2+^
DPPH (reference)	335.7		2.0036	Standard

a Indicates annealing temperatures.

**Table 3:** Percentage of Cu^+^ and Cu^2+^ impurity ions in the materials annealed at optimised temperatures. The areas under the curves of the deconvoluted peaks (932.1 and 933.4 eV) were used to determine the relative concentrations of copper species

Material annealing atmosphere	% of Cu^+^ (2p_3/2_), peak position at 932.1 eV	% of Cu^2+^ (2p_3/2_), peak position at 933.4 eV	% of O^−^ (1s), peak position at 530 eV	% of O_vac._− (1s) peak position at 531.18 eV
NaLi_2_PO_4_:Cu(i), (annealed at 600 °C)	55.40%	44.59%	72.40%	27.59%
NaLi_2_PO_4_:Cu(i), (annealed in 10% H_2_ in Ar gas at 400 °C)	38.68%	61.31%	73.50%	26.49%
NaLi_2_PO_4_:Cu(i), (annealed in CO produced by burning charcoal at 1073 K 800 °C)	100%	0.0%	66.25%	33.37%
NaLi_2_PO_4_:Cu(ii), (annealed at 400 °C)	79.53%	20.46%	38.62%	61.37%
NaLi_2_PO_4_:Cu(ii), (annealed in 10% H_2_ in Ar gas at 400 °C)	50.12%	49.87%	57.04%	42.95%
NaLi_2_PO_4_:Cu(ii), (annealed in CO produced by burning charcoal 800 °C)	100%	0.0%	60.99%	39.00%

### 3.8. XPS spectra of NaLi_2_PO_4_:Cu(ii) and NaLi_2_PO_4_:Cu(i) materials annealed in various atmospheres and temperatures

**Fig. 8:**
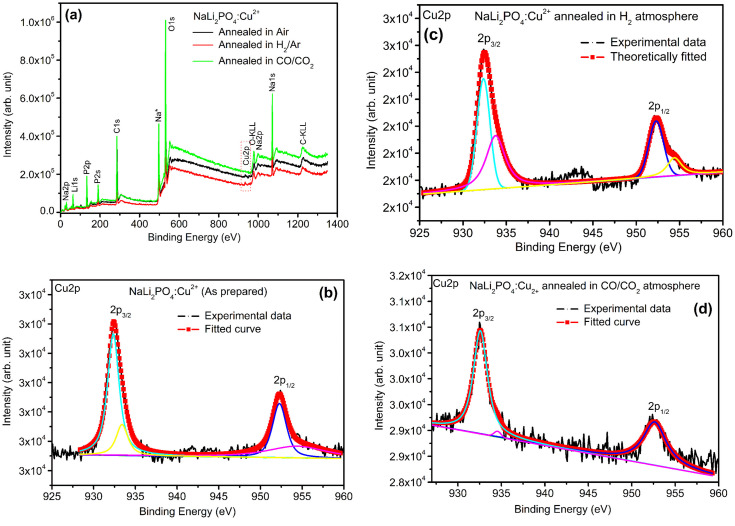
(a) XPS survey of NaLi_2_PO_4_:Cu(ii) annealed in various atmospheres for Cu ions, (b) XPS deconvoluted spectrum of NaLi_2_PO_4_:Cu(ii) as prepared sample for Cu 2p_3/2_ and Cu 2p_1/2_, (c) XPS spectrum of NaLi_2_PO_4_:Cu(ii) annealed in 10% H_2_ in Ar atmosphere at 400 °C for Cu 2p_3/2_ and Cu 2p_1/2_, (d) XPS spectrum of NaLi_2_PO_4_:Cu(ii) annealed in CO/CO_2_ atmosphere at 800 °C for Cu 2p_3/2_ and Cu 2p_1/2_.

**Fig. 9:**
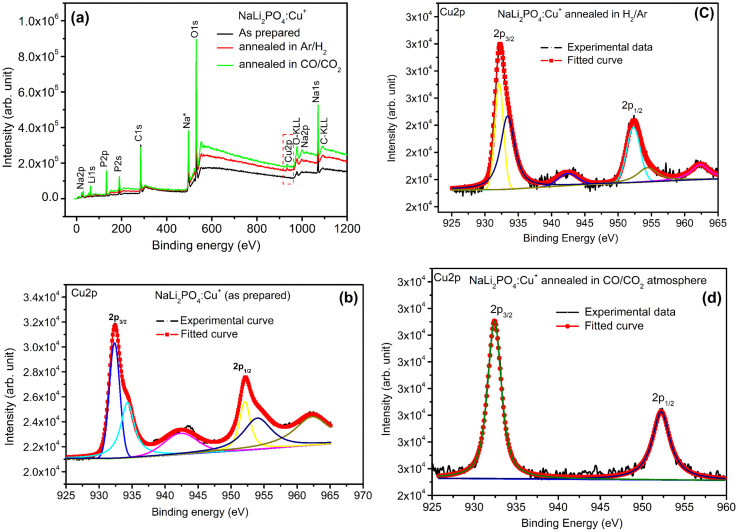
(a) XPS survey of NaLi_2_PO_4_:Cu(i) annealed in various atmospheres for Cu ions, (b) XPS deconvoluted spectrum of NaLi_2_PO_4_:Cu(i) as prepared sample for Cu 2p_3/2_ and Cu 2p_1/2_, (c) XPS spectrum of NaLi_2_PO_4_:Cu(i) annealed in 10% H_2_ in Ar atmosphere at 400 °C for Cu 2p_3/2_ and Cu 2p_1/2_, (d) XPS spectrum of NaLi_2_PO_4_:Cu(i) annealed in CO/CO_2_ atmosphere at 800 °C for Cu 2p_3/2_ and Cu 2p_1/2_.

**Fig. 10:**
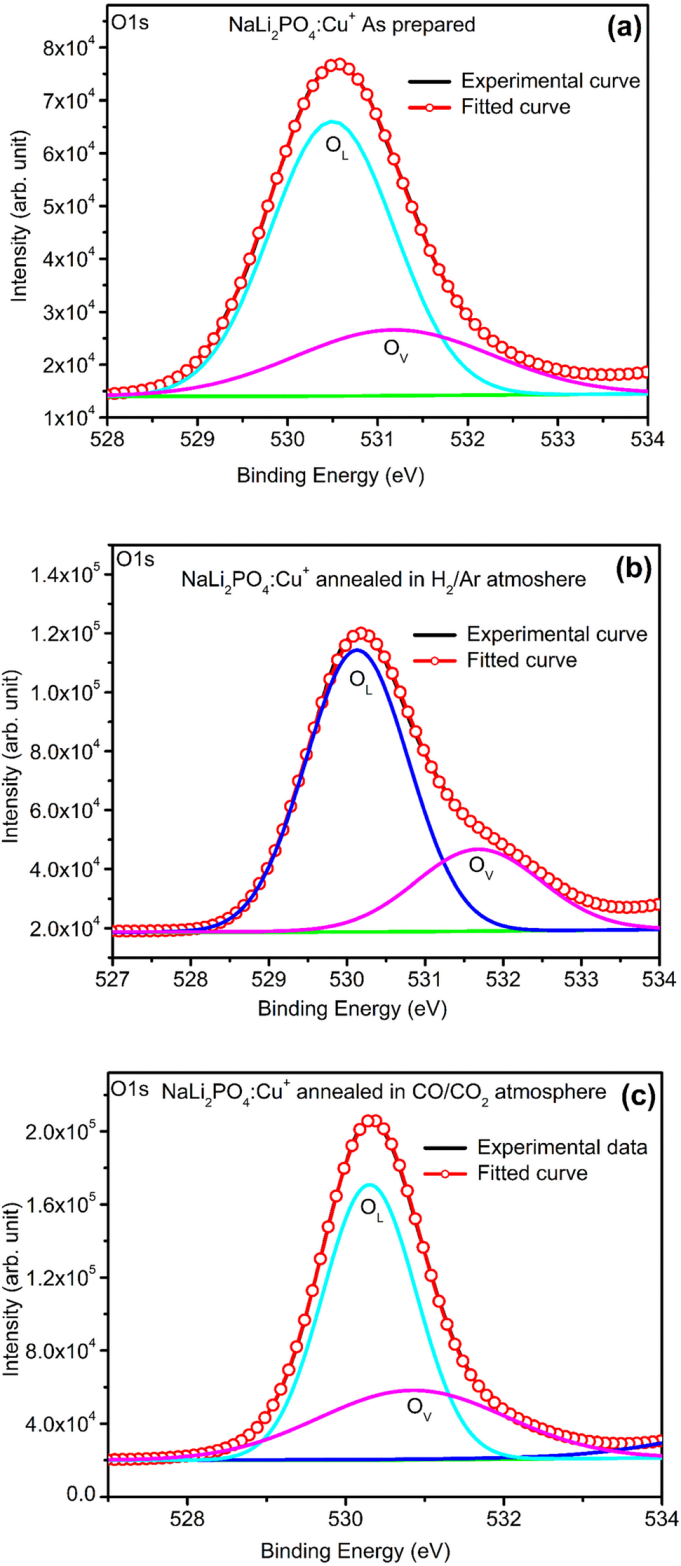
(a) XPS deconvoluted spectrum of NaLi_2_PO_4_:Cu(i) as prepared sample for O 1s and its vacancies, (b) XPS spectrum of NaLi_2_PO_4_:Cu(i) annealed in 10% H_2_ in Ar atmosphere at 400 °C for O 1s and its vacancies, (c) XPS spectrum of NaLi_2_PO_4_:Cu(i) annealed in CO/CO_2_ atmosphere at 800 °C for O 1s and its vacancies.

**Fig. 11:**
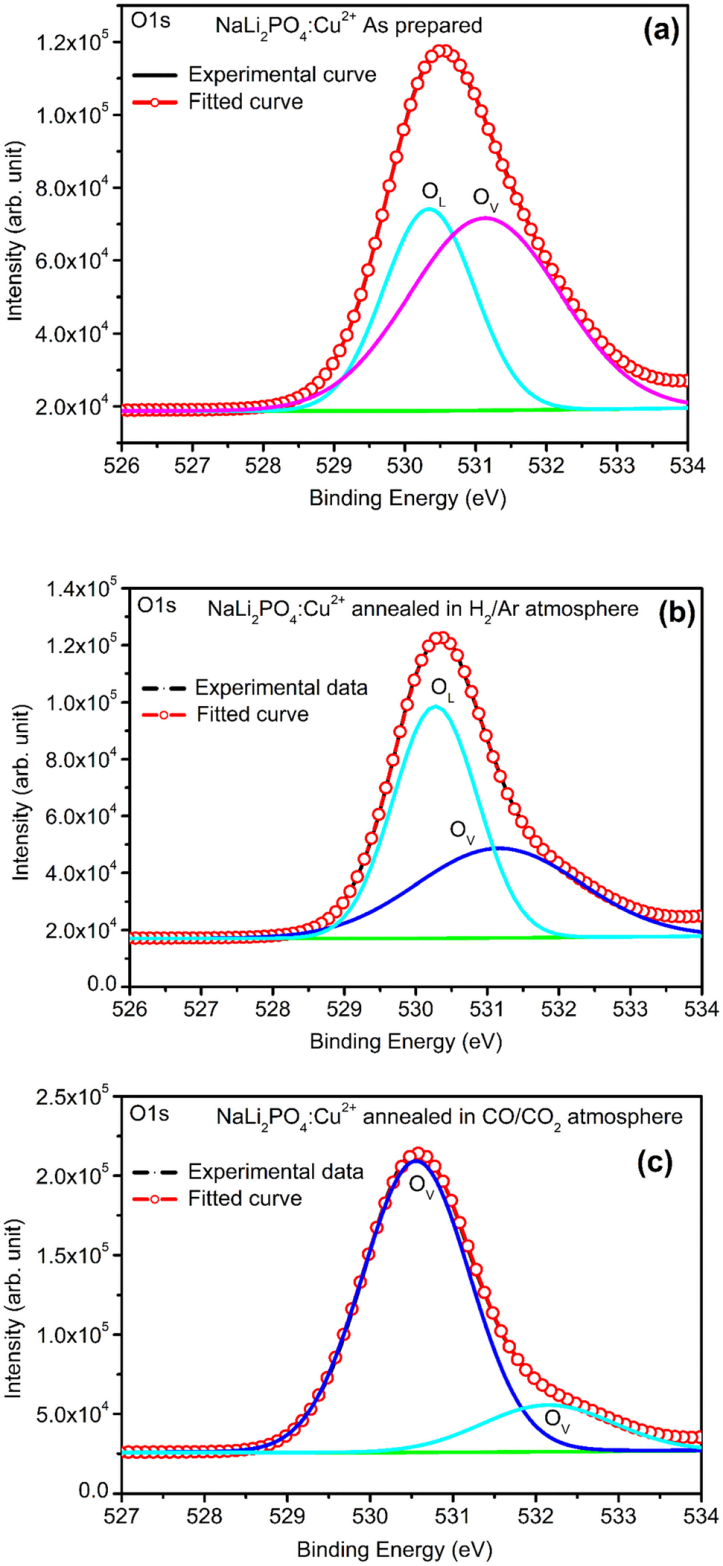
(a) XPS deconvoluted spectrum of NaLi_2_PO_4_:Cu(ii) as prepared sample for O 1s and its vacancies, (b) XPS spectrum of NaLi_2_PO_4_:Cu(ii) annealed in 10% H_2_ in Ar atmosphere at 400 °C for O 1s and its vacancies, (c) XPS spectrum of NaLi_2_PO_4_:Cu(ii) annealed in CO/CO_2_ atmosphere at 800 °C for O 1s and its vacancies.

XPS was done to know more about the composition of the material under investigation, especially, about the ionic states of the impurity ions doped in the materials and the changes taking place due to redox reactions occurring on their annealing at different oxidising and reducing atmospheres, (*i.e.*, in air, 10% H_2_ in Ar and CO/CO_2_). Though the experiments were conducted on all the materials, the results of only the materials annealed at optimized temperatures (400, 400 and 800 °C in air, 10% H_2_ in Ar and CO/CO_2_ atmospheres for NaLi_2_PO_4_:Cu(ii) and 600, 400 and 800 °C temperatures in these atmospheres, respectively) are presented here (Fig. 8 and 9). Fig. 8(a) and 9(a) show the complete XPS spectra of NaLi_2_PO_4_:Cu(ii) and NaLi_2_PO_4_:Cu(i) samples annealed in different atmosphere, respectively, over the whole energy range of 0–1200 eV. The enlarged views of the spectra in different ranges corresponding to (O 1s and Cu 2p) are also shown in Fig. 8(b–d), 9(b–d), 10(a–c) and 11(a–c) for better clarity. Different peaks are also deconvoluted using the software XPS PEAK-41 to know about the relative concentration of different species and are shown in these figures. The results are summarized in Table 3.

The ratios of areas under these curves to the total area of a particular peak after deconvolution was used to determine the relative concentration of different ionic compositions.^45^ For example, the deconvoluted spectra in the range of 925–960 eV show that the relative concentration of Cu^2+^ and Cu^+^ on annealing in different atmospheres. XPS spectra for Cu^+^ after deconvolution show peaks at around 932.1 and 952.4 eV for 2p_3/2_ and 2p_1/2_ energy levels, respectively and similarly at 933.4 eV and 954.4 eV for Cu^2+^ ions corresponding to 2p_3/2_ and 2p_1/2_ levels.^46–49^ However, although the ionic states of the impurity before doping was in Cu^2+^ (CuCl_2_) and Cu^+^ (Cu_2_Cl_2_) forms, respectively, for the materials NaLi_2_PO_4_:Cu(ii) and NaLi_2_PO_4_:Cu(i), the relative concentrations after synthesis were found to be in both Cu^+^ and Cu^2+^ (55.40 and 44.59% in pristine NaLi_2_PO_4_:Cu(i) and 79.53 and 20.46% in pristine NaLi_2_PO_4_:Cu(i)) states. After annealing in H_2_ atmosphere at 400 °C they changed to 38.68% and 61.31% for Cu^+^ and Cu^2+^ in NaLi_2_PO_4_:Cu(i) and 50.12% and 49.87% for the same in NaLi_2_PO_4_:Cu(ii), respectively, and in CO/CO_2_ atmosphere at 800 °C changed to 100% Cu^+^ ionic state in both the cases. It shows that redox reactions are occurring on annealing in oxidizing atmospheres and during synthesis also. These kind of reactions not only change the ionic states of the doped impurity but also change the concentrations of oxygen and its vacancies probably due to diffusion of atmospheric oxygen at high temperatures as shown in the table (Table 3). The diffusion of CO at such temperatures might be affecting the concentration(s) of oxygen species/vacancies. It might not only be affecting their concentrations but might also act as impurity, generate different kinds of other complex defects responsible for enhancement of TL in NaLi_2_PO_4_:Cu(ii) phosphor material. The XPS results are supporting the ESR results discussed earlier (Section 3.7).

### 3.9. Photoluminescence (PL) spectra

**Fig. 12:**
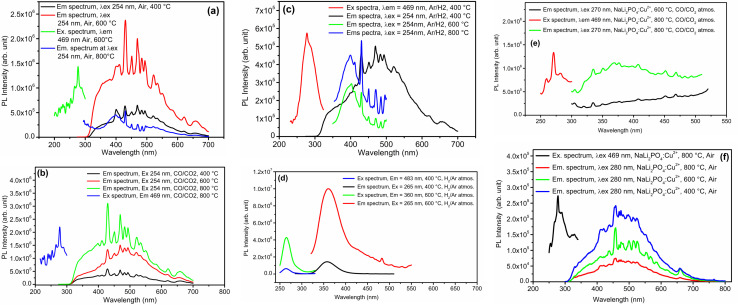
(a) PL emission and excitation spectra of NaLi_2_PO_4_:Cu(i) annealed in air atmosphere at 400, 600 and 800 °C, (b) PL emission and excitation spectra of NaLi_2_PO_4_:Cu(i) annealed in CO/CO_2_ atmosphere at 400, 600 and 800 °C, (c) PL emission and excitation spectra of NaLi_2_PO_4_:Cu(i) annealed in 10% H_2_ in Ar at 400, 600 and 800 °C, (d) PL emission and excitation spectra of NaLi_2_PO_4_:Cu(ii) annealed in 10% H_2_ in Ar at 400 and 600 °C, (e) PL emission and excitation spectra of NaLi_2_PO_4_:Cu(ii) annealed in CO/CO_2_ atmosphere at 600 and 800 °C, (f) PL emission and excitation spectra of NaLi_2_PO_4_:Cu(ii) annealed in 10% H_2_ in Ar at 400, 600 and 800 °C.

Photoluminescence (PL) emission and excitation spectra of all the samples annealed at different temperatures in different oxidizing and reducing atmospheres was recorded at room temperature. The results are given in Fig. 12 (a–d). The excitation/emission spectra shown in this figure are that of NaLi_2_PO_4_:Cu(i) annealed at different temperatures (400, 600 and 800 °C) in air, CO/CO_2_ and H_2_ in Ar, respectively. The excitation spectra (by keeping emission wavelength at around 469 nm) consists of a sharp peak at around 254 nm. This wavelength, therefore, was chosen as excitation wavelength for all the NaLi_2_PO_4_:Cu(i) samples. Emission spectrum consists of a broad band at around 350 nm and many other sharp emission at around 404, 430, 450, 469, 484, 495, 523, 534, 552, 620 and 661 nm. The 350 nm band may be attributed to 3d^9^ → 4s^1^3d^10^ transitions, while the other sharp transitions could be from different sublevels of ^2^T_2g_ → ^2^E_g_ bands of Cu^2+^. The Cu^2+^ ions belong to the 3d^9^ configuration. Depending upon its octahedral, tetragonal, and square planar (2D) configurations, the levels may split into different configurations. For example, within the octahedron crystal field, the 2D states split into ^2^E_g_ and ^2^T_2g_ energy levels. The ^2^E_g_ levels split into ^2^B_1g_ and ^2^A_1g_, and the ^2^T_2g_ levels are split into ^2^B_2g_ and ^2^E_g_. Of these levels, ^2^B_1g_ becomes the ground state. Therefore, in the case of copper ions, there are at least three bands corresponding to the transition of ^2^B_1g_ → ^2^A_1g_, ^2^B_1g_ → ^2^B_2g_ and ^2^B_1g_ → ^2^E_g_ are expected.^48–52^ However, in the present case as the redox reactions occur and as the impurity ions could occupy different positions, *i.e.*, substituting for Li^+^ or Na^+^ and getting the charge balanced the PO_4_^−^ octahedra may get very much distorted giving rise to more energy levels. Therefore, many more emissions are thus seen as expected. It could also be seen in these figures (Fig. 12(a–d)) the redox reactions occurring. For example, for the materials annealed in air (Fig. 12(a)), the overall intensity of the emission spectrum is low and the ratios of the 350 nm band of Cu^+^ some of the prominent bands of Cu^2+^ are little less than one, however, as the annealing temperature is increased to 600 °C, these bands corresponding to Cu^2+^ become more prominent, indicating that Cu^+^ → Cu^2+^ + e^−^ type of reactions are occurring. However, at still higher temperature (800 °C) more complex defects must be generated decreasing the overall intensity but the 350 nm band is still prominent. In case of the materials annealed in CO/CO_2_ atmosphere, the overall intensity goes on increasing as the temperature is increased from 400 to 800 °C, while in case of the materials annealed in H_2_ (10% H_2_ in Ar), the 350 nm band is weak initially but the temperature is increased from 400 to 800, this band becomes more prominent indicating that Cu^2+^ + e^−^ → Cu^+^ kind of redox reactions are occurring. In case of NaLi_2_PO_4_:Cu(ii) materials annealed in H_2_, the excitation and emission spectra are very much different, the excitation and emission bands were observed at around 262 and 360 nm, respectively and the intensity increased with the temperature increasing up to 600 °C. However, in cases of the as prepared as well as annealed in CO/CO_2_ materials, they are the same as in earlier cases, though the intensity of these spectra is very low as compared to the earlier ones. This is in confirmation with the XPS results that though the before doping the impurity was in Cu^2+^ state it was incorporated prominently as Cu^+^ in the host matrix (Fig. 8).

### 3.10. Comparison of sensitivity of NaLi_2_PO_4_:Cu(ii) and NaLi_2_PO_4_:Cu(i) materials with that CaSO_4_:Dy (TLD-900) commercial phosphor

**Fig. 13:**
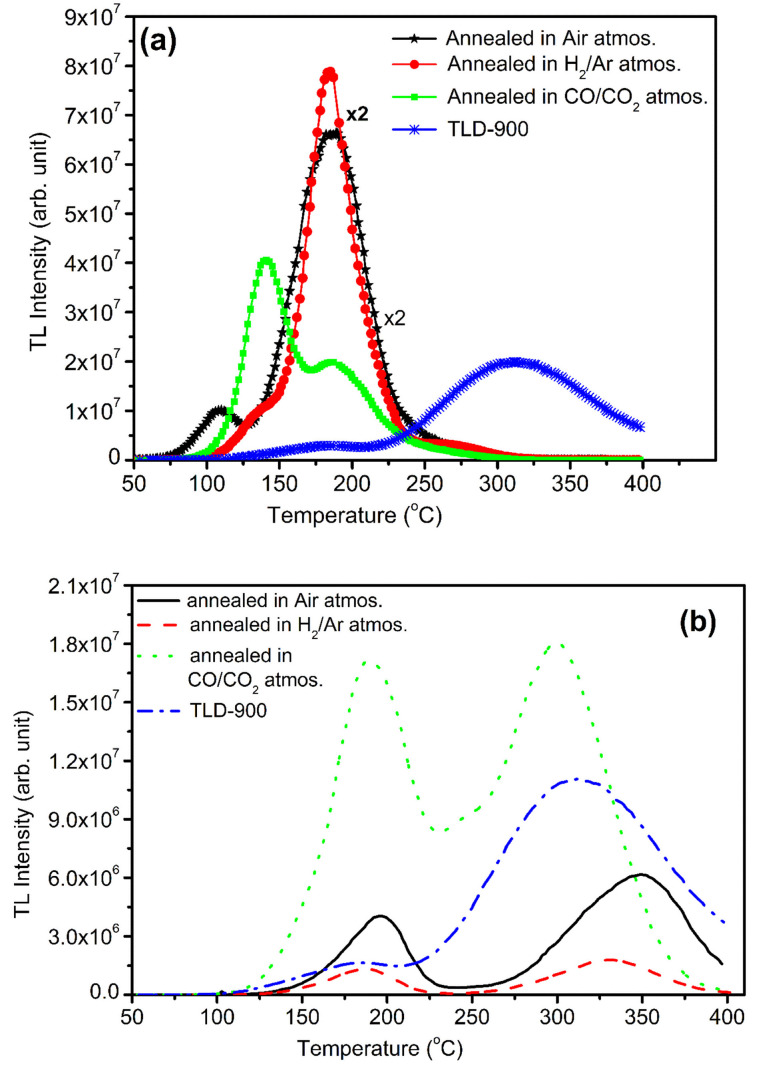
(a) Comparison of TL sensitivity of the NaLi_2_PO_4_:Cu(ii) materials annealed in air, in 10% H_2_ in Ar and in CO/CO_2_ atmospheres annealed at 400, 400 and 800 °C, respectively, with that of CaSO_4_:Dy (TLD-900) phosphor, (b) similar comparison of NaLi_2_PO_4_:Cu(i) phosphors annealed under same conditions.

TL glow curves of NaLi_2_PO_4_:Cu(ii) annealed in different atmospheres at different elevated temperatures with that of with TLD-900 are shown in Fig. 13(a). The TL intensity of NaLi_2_PO_4_:Cu(ii) annealed in air (annealed at 400 °C), in 10% H_2_ in Ar atmosphere (annealed at 400 °C) and CO/CO_2_ atmosphere (annealed at 800 °C) are 3.3, 3.0, 1.0 times more than that of CaSO_4_:Dy commercially available phosphor at 10 Gy, respectively, as shown in Fig. 13(a).

TL glow curves of NaLi_2_PO_4_:Cu(i) annealed in different atmospheres at different elevated temperatures with that of with TLD-900 are shown in Fig. 13(b). The TL intensity of NaLi_2_PO_4_:Cu(i) material annealed in air (annealed at 400 °C), in 10% H_2_ in Ar atmospheres (annealed at 400 °C) are 0.5, 0.25 times less but at least 1.8 times enhancement could be seen on annealing in CO/CO_2_ atmosphere (annealed at 800 °C) than that of CaSO_4_:Dy commercially available phosphor at 10 Gy, respectively, as shown in Fig. 13(b).

### 3.11. Dose response of the materials annealed in air atmosphere

**Fig. 14:**
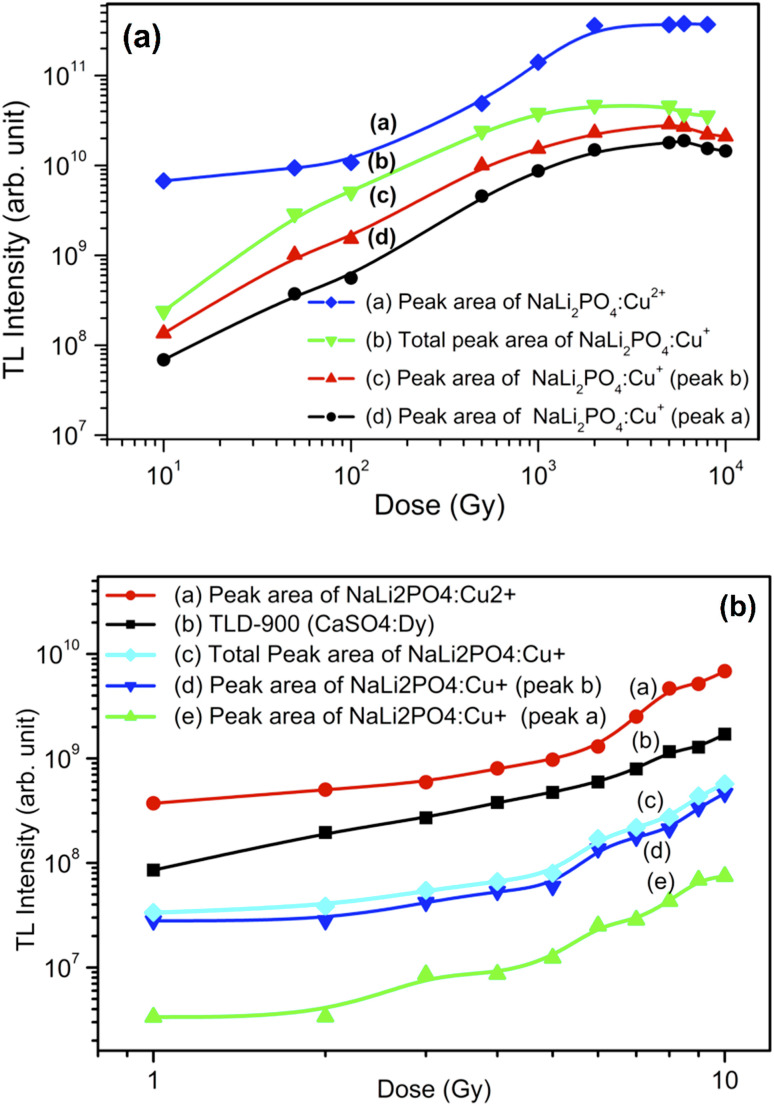
(a) Dose response (curve a) of NaLi_2_Po_4_:Cu(ii) (0.1 mol%) material annealed at 400 °C in air in the dose range of 1.0 to 10 Gy. The dose response (curve b) of CaSo_4_:Dy (TLD-900) is also given for comparison. Dose response (curve c) of NaLi_2_Po_4_:Cu(i) (0.1 mol%) material annealed at 400 °C in air in the dose range of 1.0 to 10 Gy. Dose response (curve d and e) of individual peaks (peak a and peak b) are also are also given for clarity, (b) dose response (curve a) of NaLi_2_PO_4_:Cu(ii) (0.1 mol%) material annealed at 400 °C in air in the dose range of 10.0 Gy to 10 kGy. Dose response (curve b) of NaLi_2_PO_4_:Cu(i) (0.1 mol%) material annealed at 400 °C in air in the dose range of 10.0 Gy to 10 kGy. Dose response (curve c and d) of individual peaks (peak a and peak b) are also are also given for clarity.

Fig. 14(a) shows the dose responses (areas under the curves) of NaLi_2_PO_4_:Cu(ii) (0.1 mol%) and that of NaLi_2_PO_4_:Cu(i) (0.1 mol%) the former annealed at 400 °C and the later one annealed at 600 °C (temperatures optimized for TL) in air. It could be seen that NaLi_2_PO_4_:Cu(ii) (0.1 mol%) was found to be the most sensitive than CaSO_4_:Dy (TLD-900) and also NaLi_2_PO_4_:Cu(i) (0.1 mol%). All the responses were found to be almost linear up to 5.0 Gy thereafter becomes supralinear at low doses. The material was also irradiated at high doses of γ-rays from ^60^Co radioactive source and TL was taken. The high-dose response curves are as shown in Fig. 14(b). It could be seen that if we consider individual peaks of the glow curve of NaLi_2_PO_4_:Cu(i) material it seems getting saturated around 100 Gy, however, if we consider the area under both the peaks together the dose response gets widened up to 1.0 kGy though it is little supralinear. In case of NaLi_2_PO_4_:Cu(ii) material, it is sublinear up to 500 Gy and then becomes supralinear till it saturates at around 5.0 kGy.

As mentioned earlier, NaLi_2_PO_4_:Cu(ii) phosphor material with 0.1 mol% concentration and annealed in H_2_ in Ar atmosphere at 400 °C and 600 °C. The phosphor material was found to be most sensitive at 400 °C and it can be used for dose measurements. NaLi_2_PO_4_:Cu(ii) (0.1 mol%) samples were irradiated with gamma radiation by using Co^60^ source for different dose range of 10 Gy–10 kGy. The dose response of NaLi_2_PO_4_:Cu(ii) is linear up to 5.0 kGy after that it gets saturated so that it can be used for high radiation dosimetry. Similarly, NaLi_2_PO_4_:Cu(ii) (0.1 mol%) annealed in CO/CO_2_ atmosphere at 800 °C are irradiated with dose range 10.0 Gy–10 kGy by using gamma source. The dose response of it is linear up to 5.0 kGy after that it starts saturating. The dose responses of the NaLi_2_PO_4_:Cu(i) (0.1 mol%) samples annealed in 10% H_2_ in Ar and that in CO/CO_2_ atmospheres at optimized temperatures were also studied but all the dose responses are not given here due to paucity of space and similarity with the dose responses described here for annealing in air except enhanced intensities.

### 3.12. Fading

**Fig. 15:**
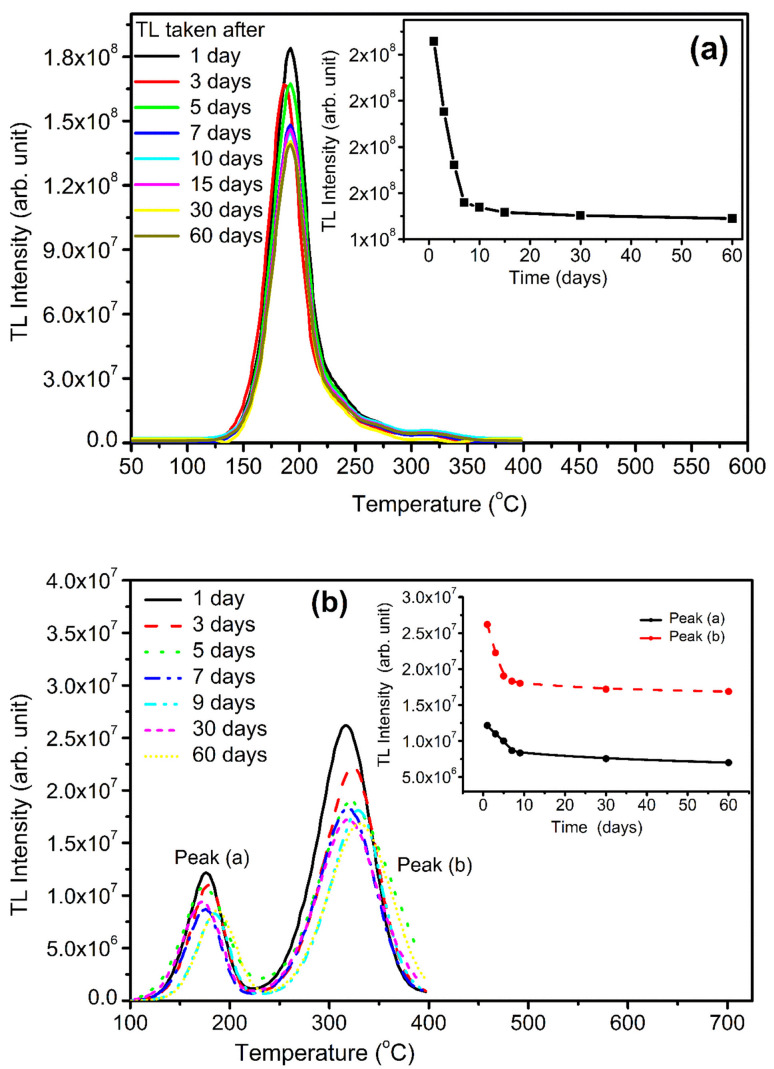
(a) TL fading of NaLi_2_PO_4_:Cu(ii), TL fading was taken immediately after the irradiation, *i.e.*, day 1 to 60 days, (b) TL fading NaLi_2_PO_4_:Cu(i) from day 1 to 60 days.

To study the fading effect (how match TL signal fad with respect to time) of the material, irradiation of 10.0 Gy of gamma ray Co^60^ source was given to the samples. The samples were stored at 300 K in the black paper to protect from the direct sunlight to stop the fast fading. In case of NaLi_2_PO_4_:Cu(ii) fading will be observed 29% shown in Fig. 15(a) and NaLi_2_PO_4_:Cu(i) fads 30% shown in Fig. 15(b) in 2 months, respectively.

### 3.13. Reusability

Reusability is one of the most important properties to satisfy for a good TLD phosphor. A material is not considered to be a good TL dosimeter, if its sensitivity/glow curve structure change after its use, *i.e.*, these characteristics should remain unaltered even after its irradiation and TL readouts for several number of cycles. Therefore, the newly developed NaLi_2_PO_4_:Cu(ii) TLD phosphor was tested for its reusability. Pallets of the samples were exposed to 10 Gy of γ-rays and TL glow curves were recorded up to 400 °C, followed by quickly cooling it to the room temperature. The same sample was annealed at 400 °C for 10 min to ensure that all traps from earlier irradiation were emptied. The sample was again irradiated second time with the same amount of dose and readout was taken using the same heating and cooling procedure. Several such cycles of irradiations and readouts were performed on the same sample by keeping all other parameters constant. No significant changes have been observed in the TL sensitivity of the phosphor material. A very good reusability is thus seen in the newly developed TLD phosphor.

## 4. Conclusions

NaLi_2_PO_4_ material was doped with two different compounds Cu_2_Cl_2_ and CuCl_2_ as sources of the Cu-impurity in order to see the effect of Cu^+^ and Cu^2+^ on the luminescence and other characteristics of the material under investigation. However, it was observed that though the initial ionic states of the impurity were Cu^+^ or Cu^2+^ they were incorporated in both the forms in the material. The impurity concentrations in both the cases were optimized and found that it gives the maximum TL intensity at around 0.1 mol%. From our earlier studies,^30^ it was known that redox reactions could occur in Cu-doped materials. Therefore, the materials were annealed in oxidizing (air) and reducing (10% H_2_ in Ar and CO/CO_2_) atmospheres. Redox reactions of Cu^0^ ↔ Cu^+^ ↔ Cu^2+^ kinds take place on annealing the materials at different temperatures and on irradiation. The redox reactions were studied using different experimental techniques, such as ESR, XPS and PL. The effect of such redox reactions on TL was also studied from dosimetry applications point of view and found that there was many fold enhancement in both Cu^+^ and Cu^2+^ doped materials. It was also found from ESR measurements that there could be incorporation of carbon due to annealing in CO/CO_2_ atmosphere at high temperatures and may act as co-dopant, form some more complex defects enhancing the TL intensity. Thus, higher sensitivity (3.3 and 1.0 times more) than the commercially available CaSO_4_:Dy (TLD-900) phosphor at optimized annealing temperatures (400 °C for annealed in 10% H_2_ in Ar and 800 °C for annealed CO/CO_2_ atmospheres) at 10.0 Gy dose, wide dose response up to 5.0 kGy, (though for NaLi_2_PO_4_:Cu(ii) materials it is sublinear up to 3.0 kGy and becomes supralinear till it gets saturated), low fading (30% in 2 months) and very good reusability make this material a good candidate for the dosimetry of high-energy radiations.

## Abbreviations

TL: Thermoluminescence
TLD: Thermoluminescence dosimetry/dosimeter
OSL: Optically stimulated luminescence
OSLD: Optically stimulated luminescence dosimetry/dosimeter
PXRD: Powder X-ray diffraction
XPS: X-ray photoluminescence spectroscopy
ESR: Electron spin resonance
PL: Photoluminescence
LC: Luminescence centre
RT: Room temperature
LED: Light emitting diode
DMFC: Digital mass flow controller
NaLi_2_PO_4_:Cu(i): Sodium lithium phosphate doped with Cu_2_Cl_2_
NaLi_2_PO_4_:Cu(ii): Sodium lithium phosphate doped with CuCl_2_

## Author contributions

Both the authors have equal contribution.

## Conflicts of interest

There is no conflict of interest to declare.

## Supplementary Material
